# Supplementary material for: Cortex-wide topography of 1/f-exponent in Parkinson’s disease
Source: NPJ Parkinsons Dis. 2023 Jul 13;9:109. doi: 10.1038/s41531-023-00553-6 (PMC10338503; doi:10.1038/s41531-023-00553-6)
Supplement: Supplementary file 1 — Supplementary Information [file 41531_2023_553_MOESM1_ESM.pdf]

Supplementary Material : Cortex-wide topography of 1/f-exponent in Parkinson's disease

Pascal Helson * [1], Daniel Lundqvist[2], Per Svenningsson[3], Mikkel C. Vinding*[2,4], and Arvind Kumar*[1]

[1]*School of Electrical Engineering and Computer Science, KTH Royal Institute of Technology, Stockholm, Sweden and Science for Life Laboratory, Sweden*

[2]*Department of Clinical Neuroscience, NatMEG, Karolinska Institutet, Stockholm, Sweden*

[3]*Section of Neurology, Department of Clinical Neuroscience, Center for Molecular Medicine, Karolinska Institutet, Stockholm, Sweden. Karolinska Hospital, Stockholm, Sweden*

[4]*Copenhagen University Hospital - Hvidovre, Copenhagen, Denmark*

*Corresponding authors: pashel@kth.se, mikkelcv@drcmr.dk, arvkumar@kth.se

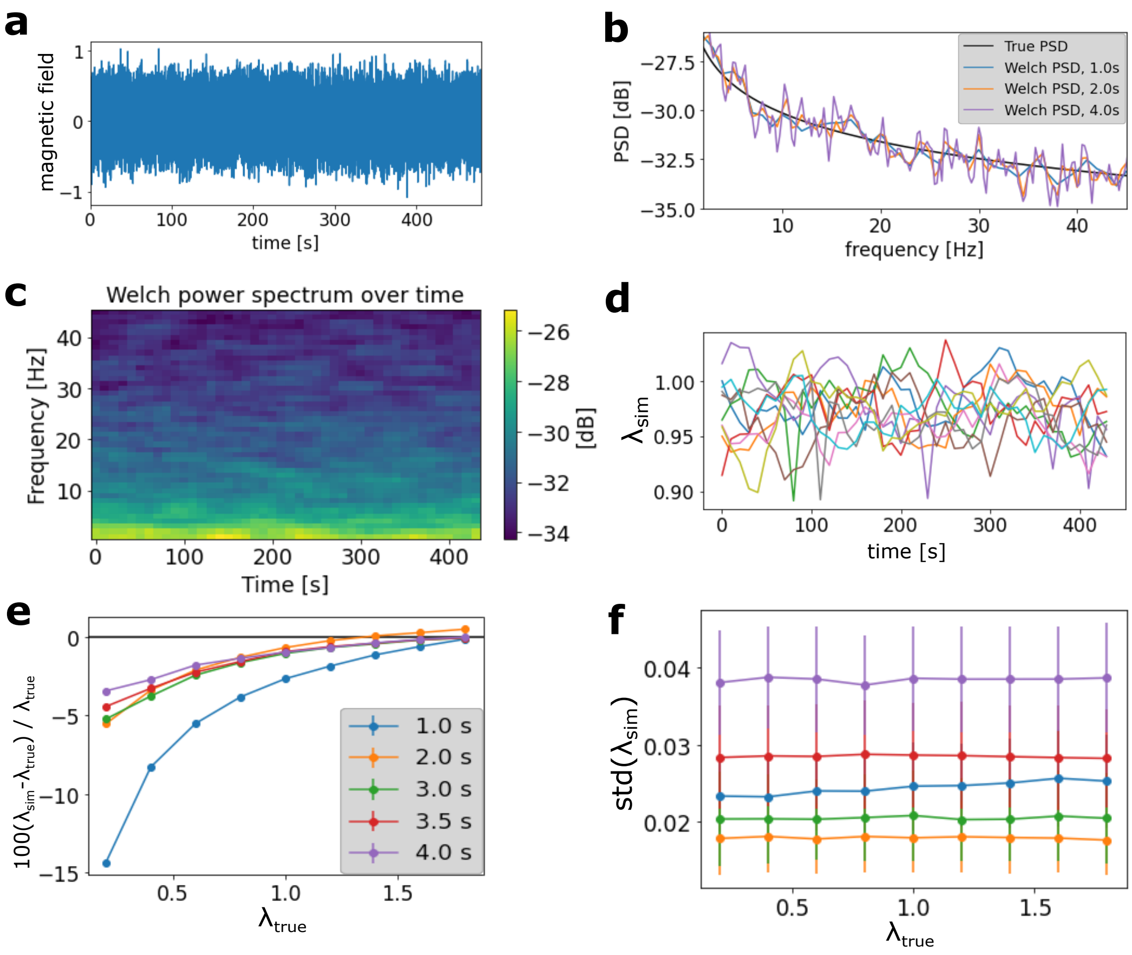

Supplementary Figure 1: Comparing the true 1/f-exponent of a simulated signal reconstructed from a given power spectrum of the form κ/f^{λ} **(a)** Reconstructed signal from PSD equals to $10^{-2.5}/f^{0.5}$. **(b)** PSD of the reconstructed signal using the entire signal or a segment of it of sizes 1, 2 or 4 sec. **(c)** Spectrogram of the reconstructed signal shown in **A** using the method described in Methods. **(d)** Temporal dynamics of λ_{sim} the reconstructed signals for a PSD $10^{-2.5}/f^{1}$ and epochs of 1 sec. **(e)** Temporal average of λ from reconstructed signals, λ_{sim}, compared to the true λ_{true}. **(f)** Temporal standard deviation of λ from reconstructed signals in function of the true λ.

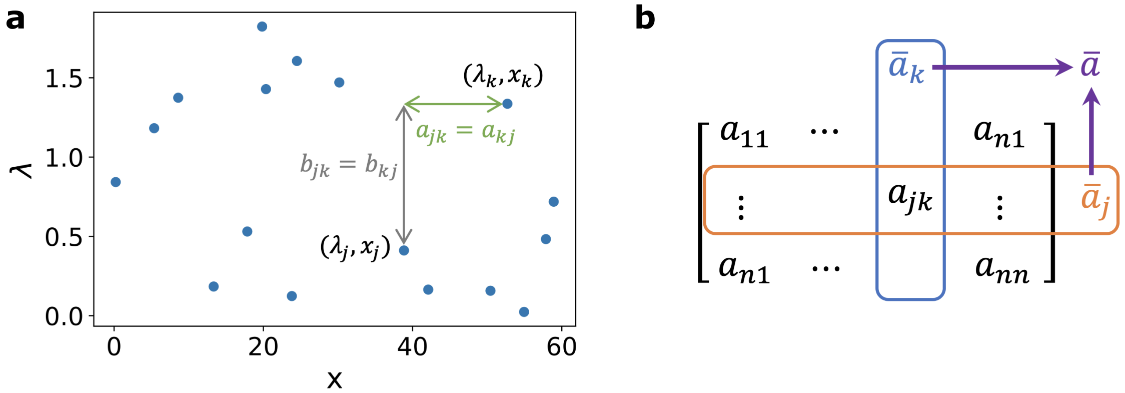

Supplementary Figure 2: Schematic of distance correlation measurement. (a) Example of a sample of (λ, x) in a given brain region for 17 different individuals (uniformly drawn). $(a_{ml})_{m,l}$ (resp. $(b_{ml})_{m,l}$) is the matrix of distances within the vector λ (resp. x). **(b)** Computations on $(a_{ml})_{m,l}$ rows and columns: $\overline{a}_j$ is the mean of the jth row (or column as $(a_{ml})_{m,l}$ is symmetric) and $\overline{a}$ is the mean of $(\overline{a}_j)_j$.

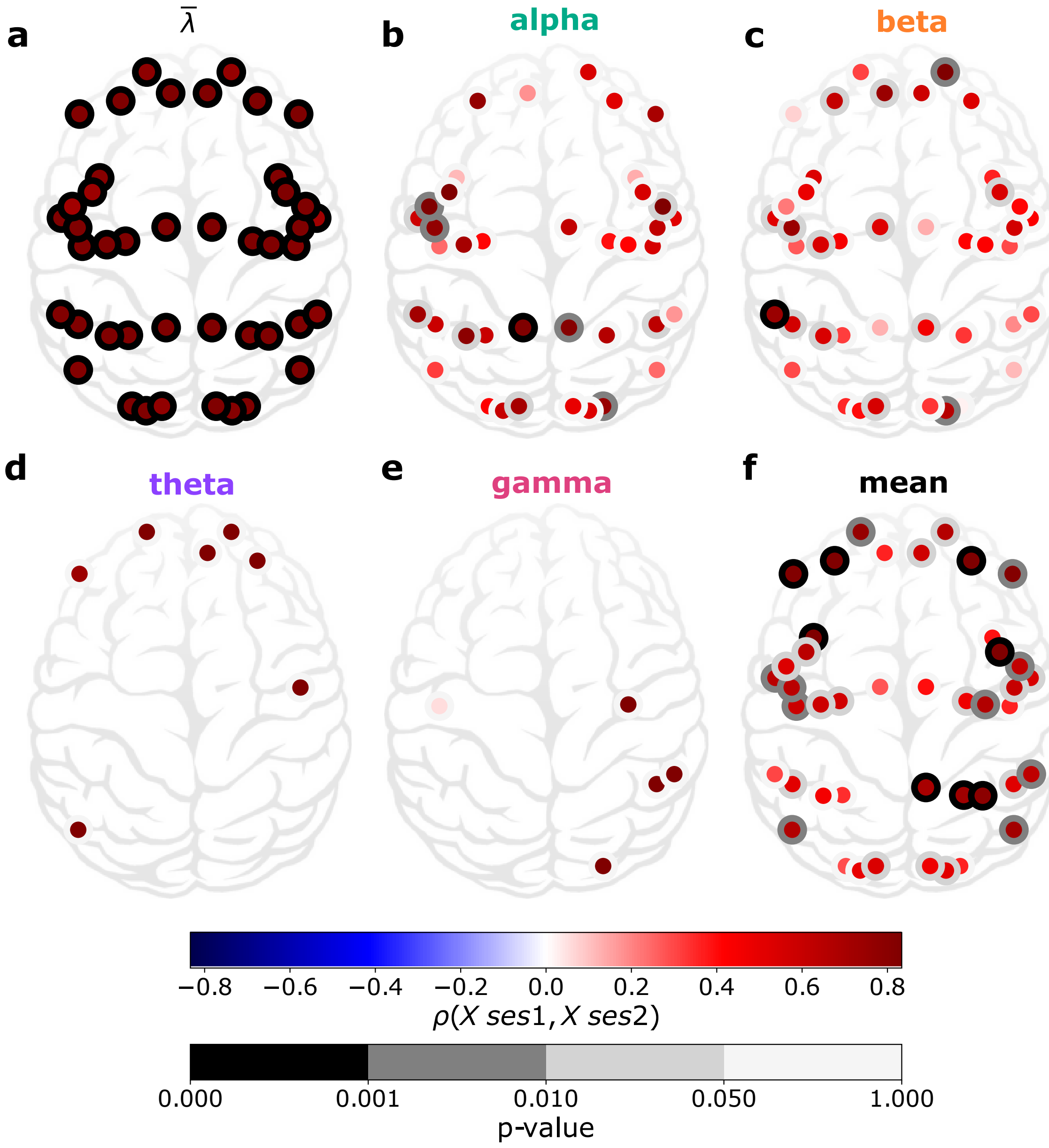

Supplementary Figure 3: Pearson correlation for test-retest reliability between sessions 1 and 2 for HCs and different parameters. Pearson correlation between : **(a)** $\overline{\lambda}_{HC\ ses1}$ and $\overline{\lambda}_{HC\ ses2}$. **(b-f)** HC session 1 and session 2 frequency peaks averaged over the different bands **(b-e)** and over all bands **(f)**.

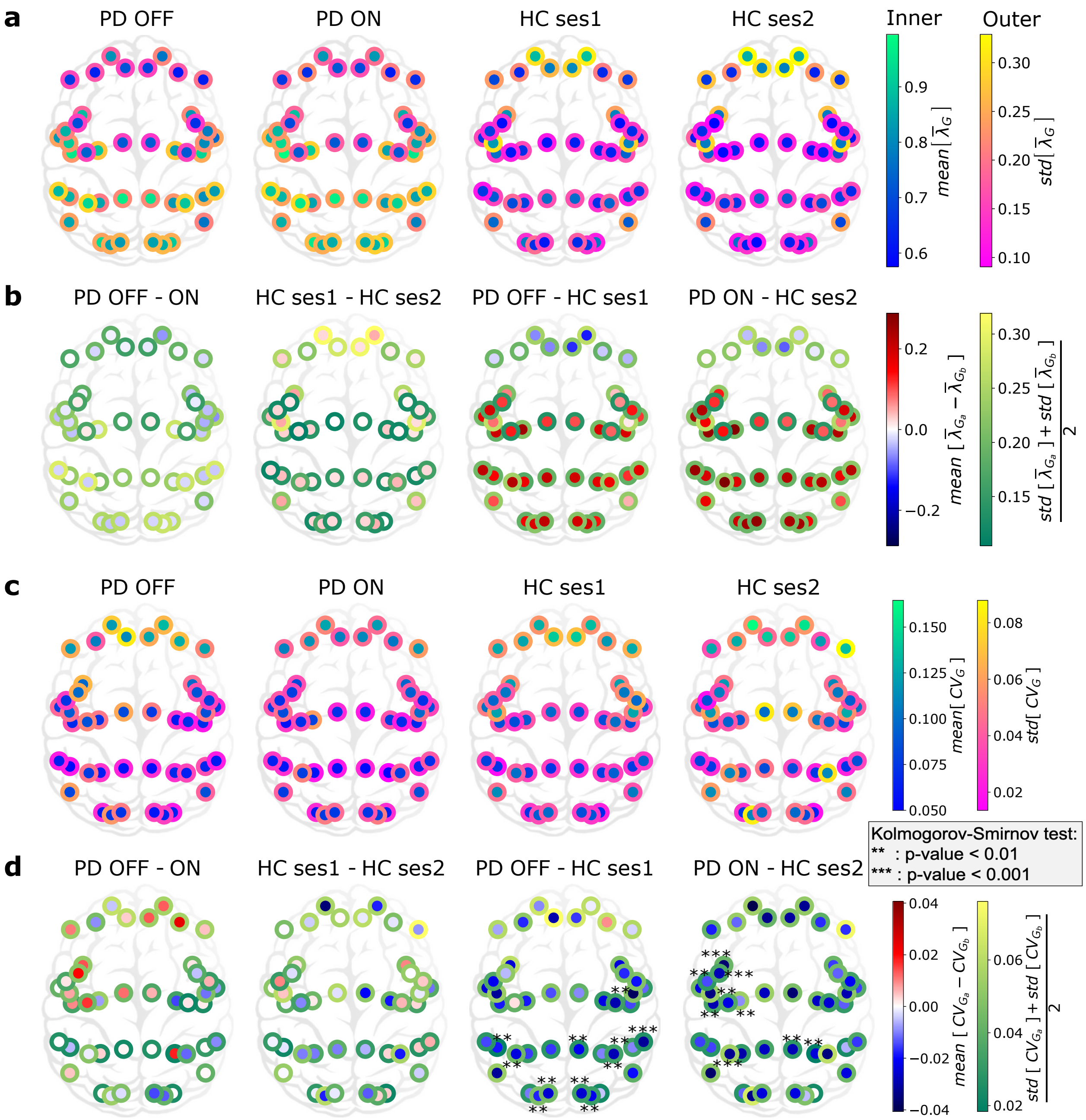

Supplementary Figure 4: Cortex-wide distribution of the mean and the coefficient of variation over time of λ within each group. **(a)** (resp. **(c)**) Temporal mean (resp. coefficient of variation) of λ_G for each group $G \in \{\text{HC ses1, HC ses2, PD-ON, PD-OFF}\}$. The inside colour refers to the mean over the group. The border colour refers to the fluctuation of $\overline{\lambda}_G$ (resp. CV_{λ_G}) within the group G. **(b)** (resp. **(d)**) Temporal mean (resp. coefficient of variation) of λ between two groups $G_a, G_b \in \{\text{HC ses1, HC ses2, PD-ON, PD-OFF}\}$. The inside colour refers to the difference between the means over the groups. The border colour refers to the averaged fluctuation of $\overline{\lambda}_G$ (resp. CV_{λ_G}) combining the two groups G_a and G_b.

Brain region \ Patient	PD ON lh	PD ON rh	PD OFF lh	PD OFF rh
AC-MPC	0.052655	0.0327	0.023435	0.05004
AAC	0.005475	0.008165	0.001995	0.01079
DSVC	0.00098	0.10805	0.001665	0.05319
DPC	0.029015	0.09365	0.020805	0.138505
EAC	0.00134	0.00813	0.00047	0.002055
EVC	0.009255	0.00509	0.017115	0.016085
IFC	0.0169	0.11816	0.065705	0.072345
IPC	0.004815	0.014195	0.00435	0.006125
I-FOC	0.001375	0.00364	0.00146	0.002615
LTC	0.00621	0.00102	0.0191	0.01329
MT+C-NVA	0.00308	0.0098	0.004235	0.00613
MTC	0.001795	0.004075	0.008265	0.00591
O-PFC	0.05662	0.15312	0.061315	0.053295
PL-MCC	0.0401	0.02616	0.09309	0.02303
PCC	0.00078	0.007705	0.00162	0.006045
POC	0.015555	0.04716	0.012155	0.02366
PMC	0.018335	0.100665	0.12863	0.05784
PVC-V1	0.01139	0.00877	0.024695	0.00548
SMC	0.025775	0.033375	0.10384	0.02987
SPC	0.001025	0.0362	0.003105	0.014585
T-P-O-J	0.000495	0.006065	0.002675	0.0031
VSVC	0.00903	0.001855	0.00938	0.009635

Supplementary Table 1: P-values of the distance correlation per brain region, PD patient session and hemisphere. The shadowed cells are the 20 lowest p-values.

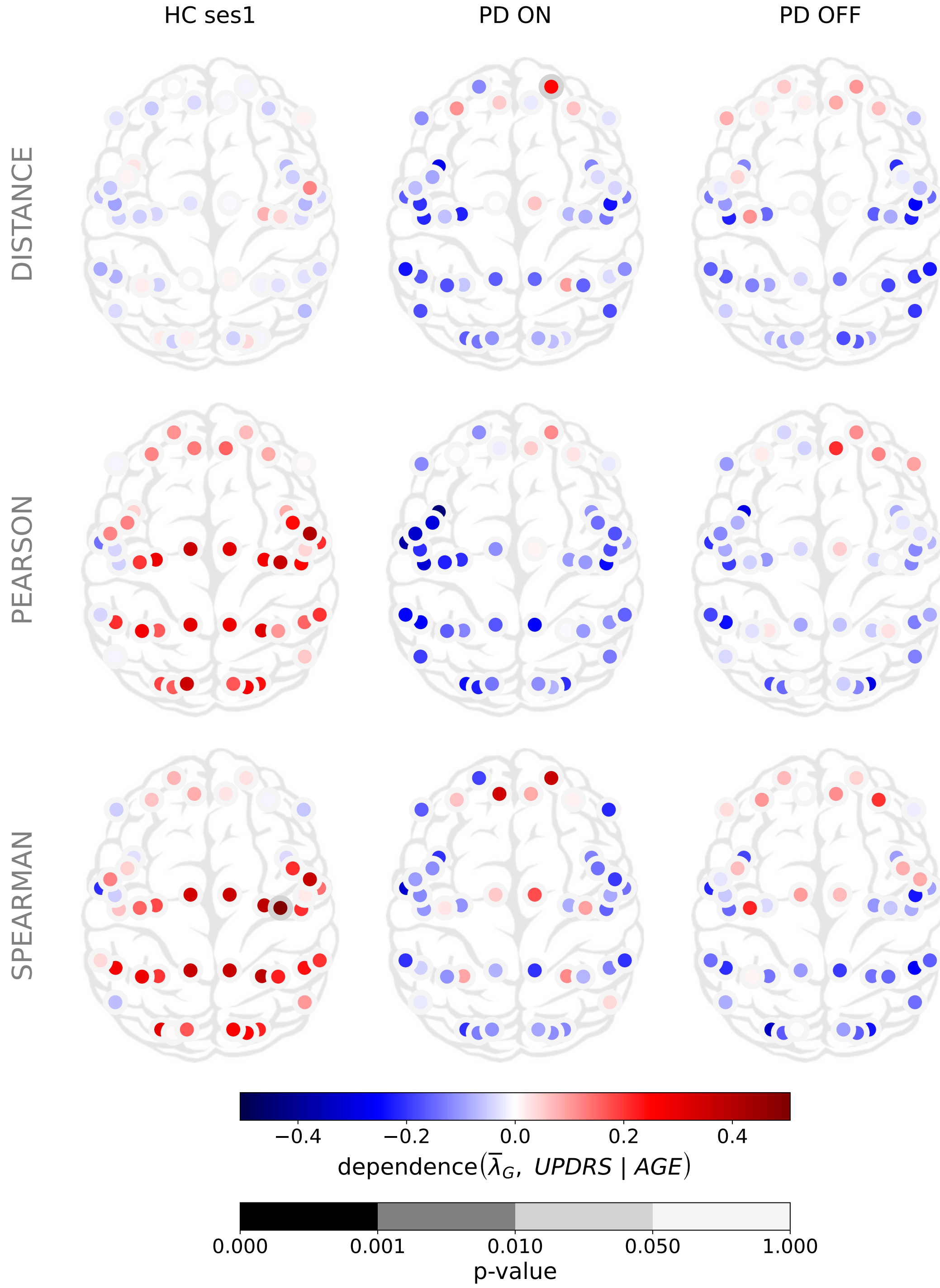

Supplementary Figure 5: Cortex-wide distribution of different relationship measures between the mean over time of λ and UPDRS-III within each PD group and combining them (first column).

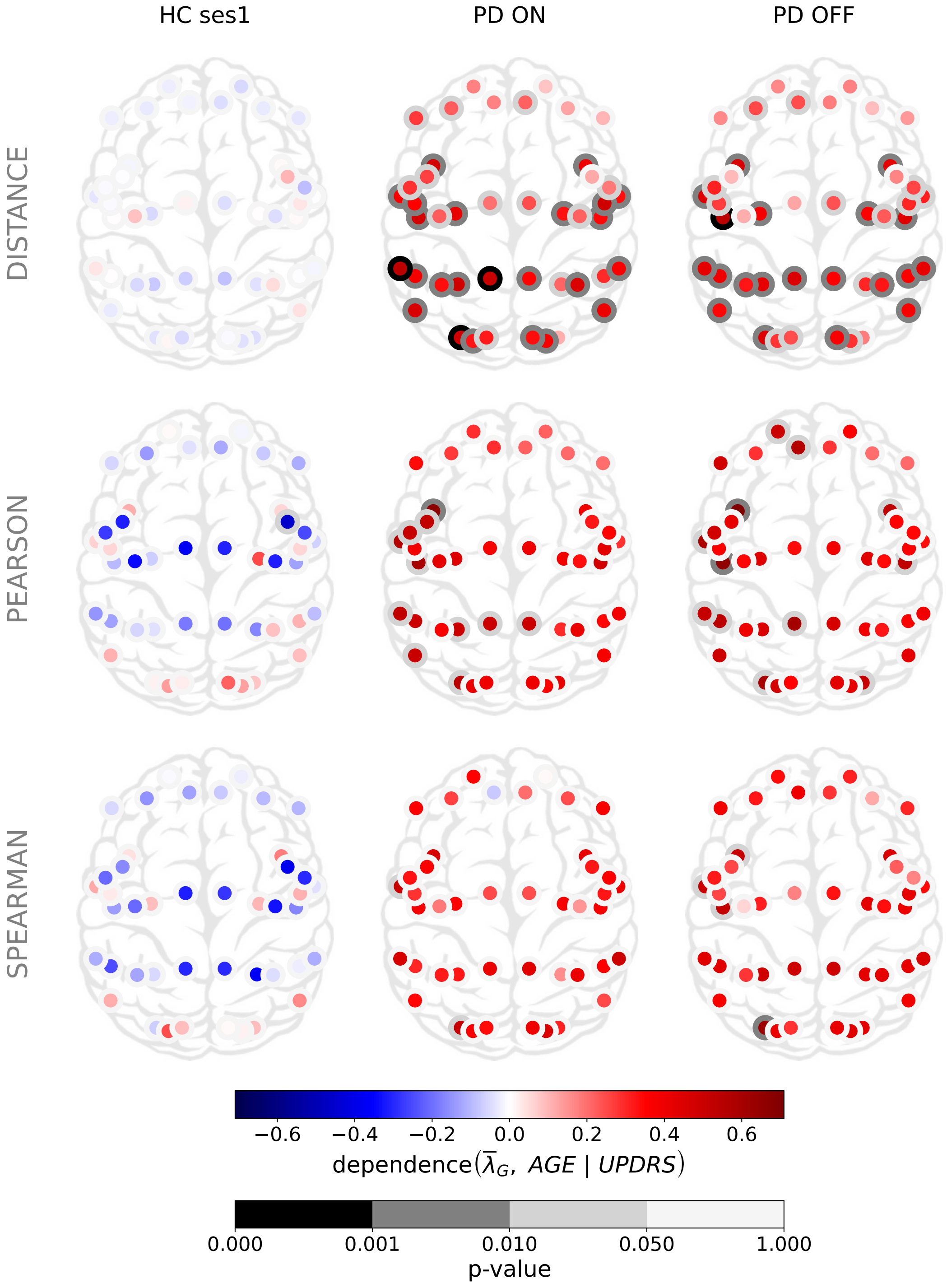

Supplementary Figure 6: Cortex-wide distribution of different relationship measures between the mean over time of λ and ages within each group.

VARIABLE / GROUP	Age (year)	Levodopa dose (mg)	Disease duration (year)	HY stage	UPDRS-III						
					F1 Gait	F2 R tremor	F3 Rigidity	F4-5 Hand	F6 P-K tremor	F7 Legs	TOTAL
HC	69,4 ± 5,7	NA	NA	0	0,4 ± 0,8	0,1 ± 0,4	0,2 ± 0,5	0,6 ± 1	0,2 ± 0,4	0,1 ± 0,4	1,6 ± 2,4
PD ON	67,2 ± 9,4	633,7 ± 280	5,3 ± 3,9	2,3 ± 0,6	4,9 ± 2,9	1,6 ± 2,6	2,2 ± 1,3	4,9 ± 3,5	2,3 ± 1,9	1,6 ± 1,5	17,5 ± 9,6
PD OFF					8,7 ± 3,9	4,1 ± 4,3	4,9 ± 2,1	8,4 ± 3,8	3,4 ± 2,6	3,8 ± 2,8	33,2 ± 11,6

Supplementary Table 2: PD patient and HC details. This table indicates the mean ± standard deviation of the different participant information. Hoehn and Yahr (HY) stage is a measure of PD progress.[1] R (resp. P-K) tremor means resting (resp. postural and kinetic) tremor. For more details on MDS-UPDRS-III subscales (F1-7) we refer to Goetz et al.[2].

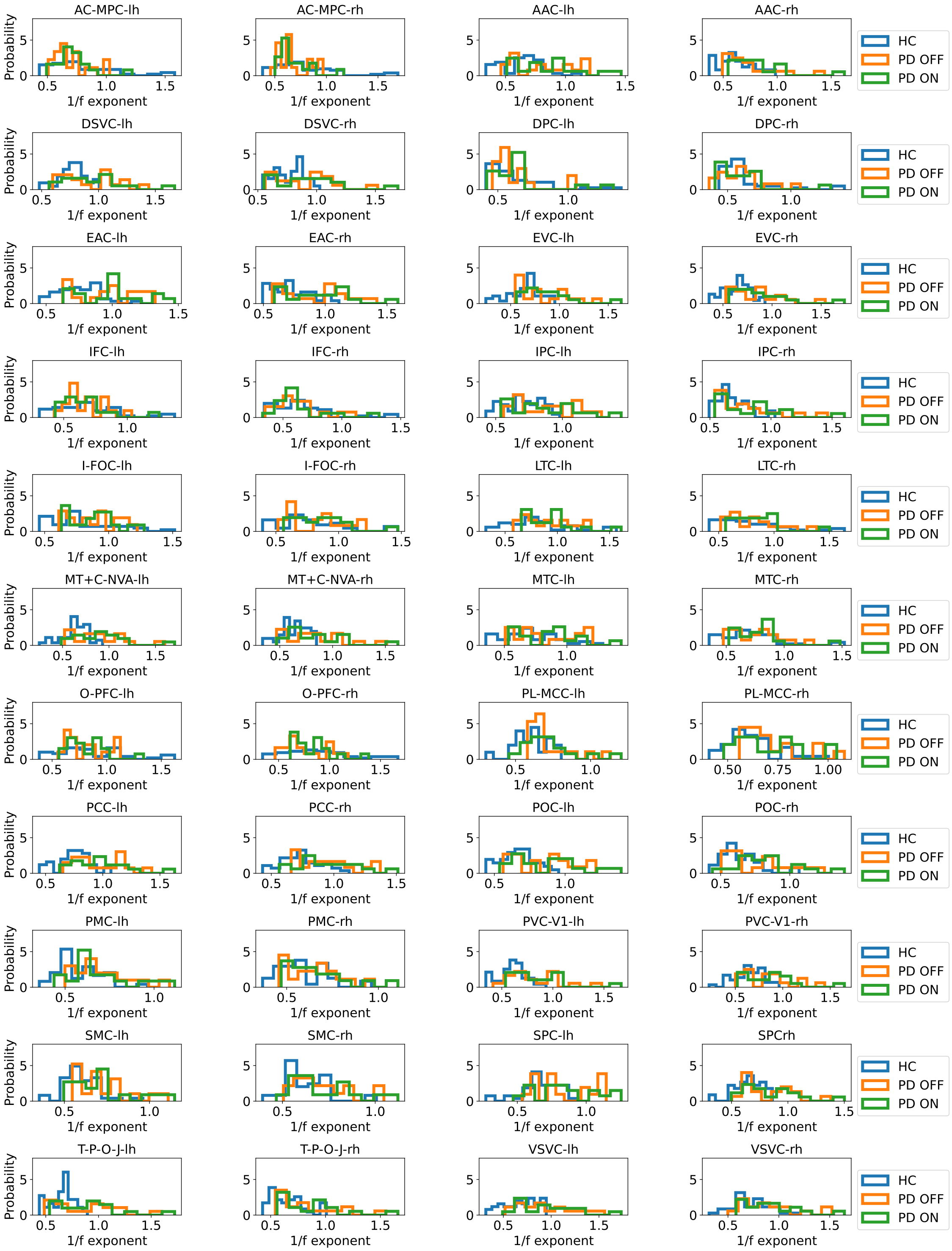

Supplementary Figure 7: 1/f-exponent distributions over the different groups in the different brain regions.

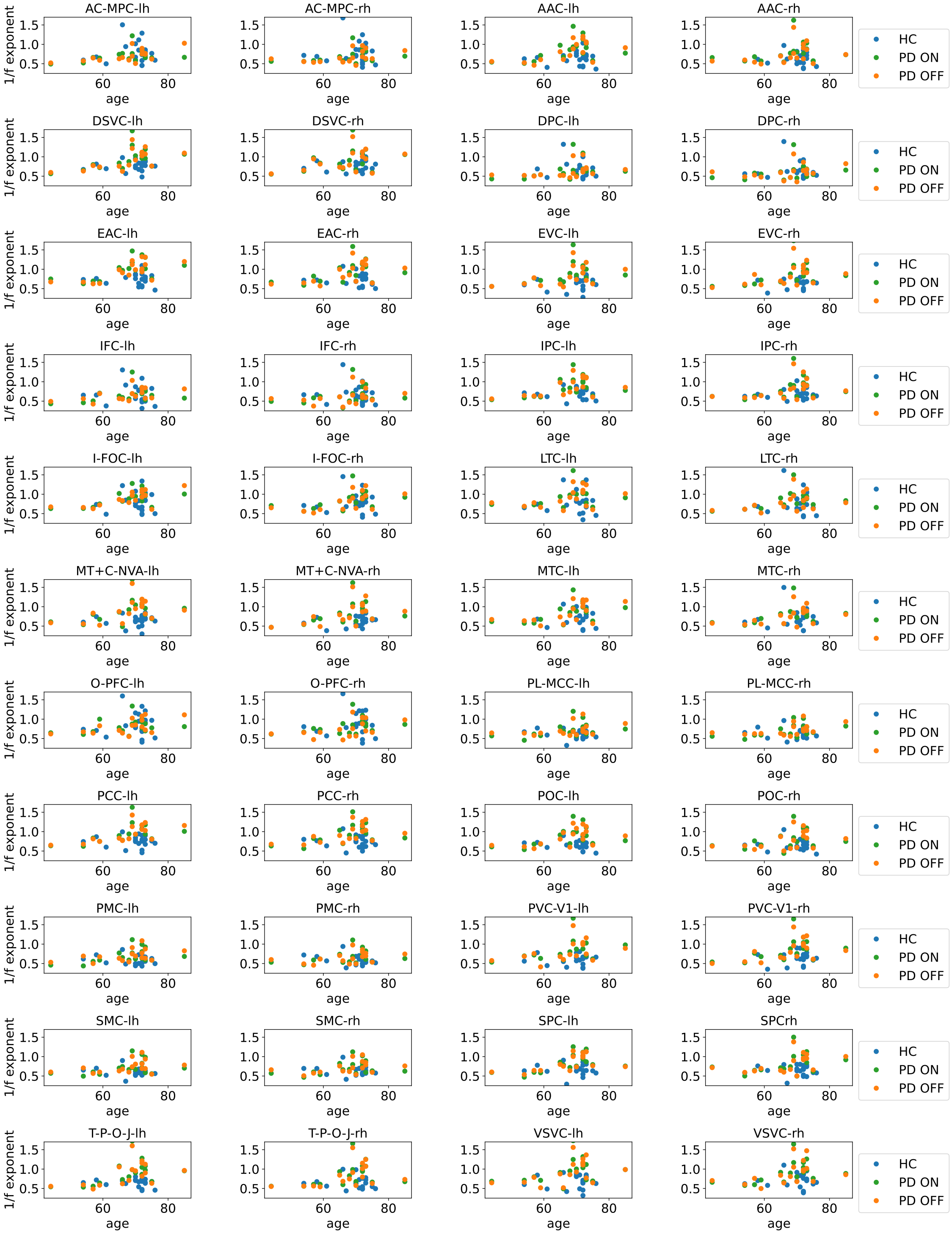

Supplementary Figure 8: 1/f-exponent as a function of age for the different groups in the different brain regions.

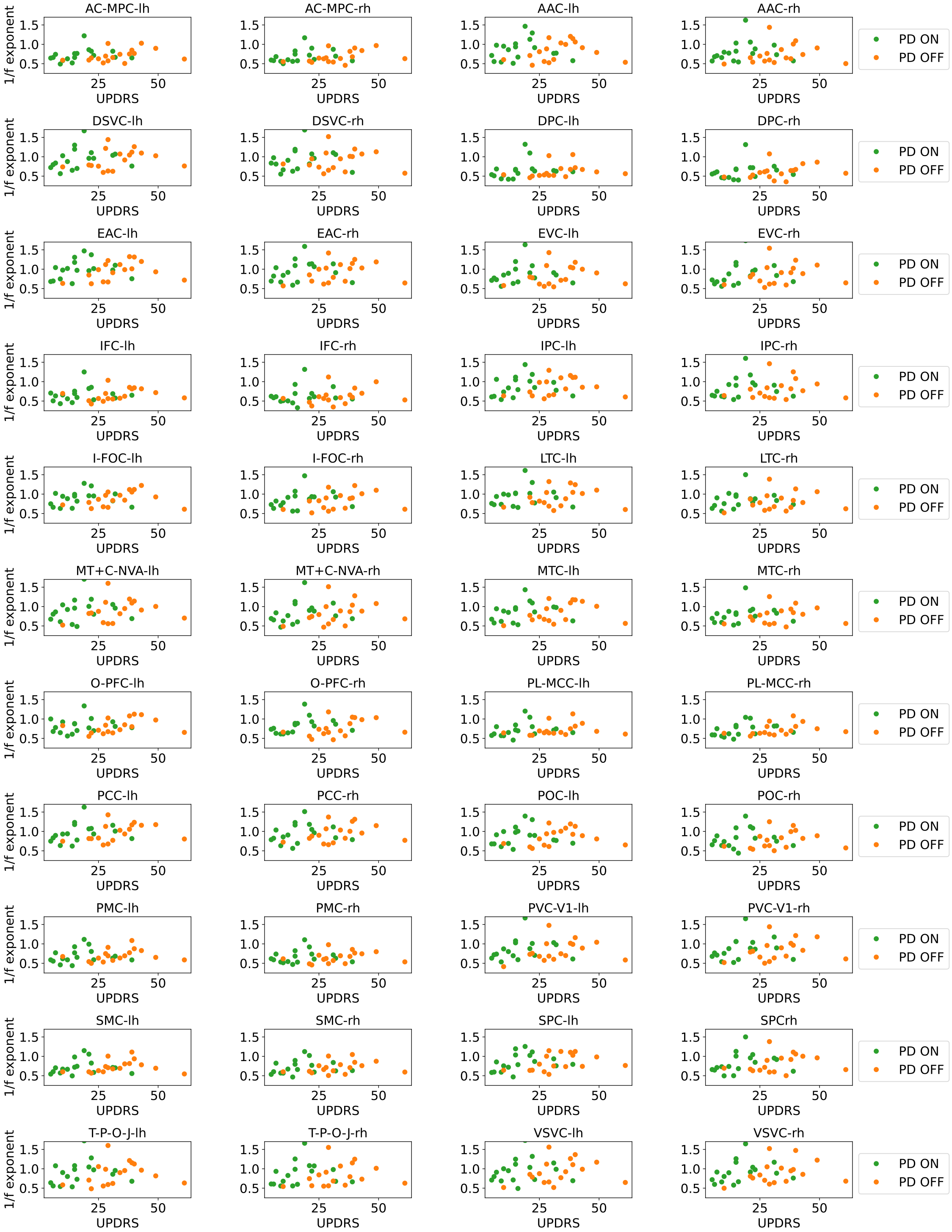

Supplementary Figure 9: 1/f-exponent as a function of UPDRS-III for PD-ON and PD-OFF in the different brain regions.

References

[1] M. M. Hoehn, M. D. Yahr, et al. “Parkinsonism: onset, progression, and mortality”. *Neurology* 50.2 (1998).

[2] C. G. Goetz, B. C. Tilley, S. R. Shaftman, et al. “Movement Disorder Society-sponsored revision of the Unified Parkinson’s Disease Rating Scale (MDS-UPDRS): Scale presentation and clinimetric testing results: MDS-UPDRS: Clinimetric Assessment”. *Movement Disorders* 23.15 (2008).
